# Effect of Institutional Trust on Consumers’ Health and Safety Perceptions and Repurchase Intention for Traceable Fresh Food

**DOI:** 10.3390/foods10122898

**Published:** 2021-11-23

**Authors:** Edward Shih-Tse Wang, Hung-Chou Lin, Ming-Chie Tsai

**Affiliations:** 1 Graduate Institute of Bio-Industry Management, National Chung Hsing University, 250, Kuo Kuang Rd., Taichung 402, Taiwan; Shihtse.wang@msa.hinet.net (E.S.-T.W.); mingchietsainchu@gmail.com (M.-C.T.); 2 Department of Adult & Continuing Education, National Taiwan Normal University, 162, Section 1, Heping E. Rd., Taipei City 106, Taiwan

**Keywords:** food traceability, fresh food, institutional trust, food safety, food health benefit, purchase intention

## Abstract

Numerous food safety incidents have gained public attention and motivated consumers to seek safer and healthier products. Some governments have responded by enacting legislation to regulate the traceability of agricultural products and enhance food safety. To elucidate factors that affect consumers’ health and safety perceptions and repurchase intention for certified traceable fresh food, this study applied institutional trust theory to explore the effects of institutional trust (i.e., trust in government, certification organizations, producers, and retailers) on consumers’ food safety and health perceptions and repurchase intention. This study was conducted in Taiwan and enrolled 393 consumers who purchased certified traceable fresh food as survey participants. Structural equation modeling and multiple and stepwise regression analysis were performed for data analysis. The results indicated that trust in government, certification organizations, food producers, and food retailers was positively related to food safety perception; trust in certification organizations, food producers, and food retailers directly influenced food healthiness perception, whereas trust in government did not have a direct influence. Furthermore, trust in certification organizations and food producers influenced repurchase intention, whereas trust in government and food retailers did not. Based on these results, the current study provides some practical suggestions for traceable fresh food marketers to use institutional trust to improve consumers’ food health and safety perceptions and repurchase intention.

## 1. Introduction

Consuming varied fresh foods affords many health benefits [1], and eating fresh fruits and vegetables is vital to maintaining a healthy lifestyle [2]. However, a series of food safety scandals beginning in the late 1990s eroded consumer confidence in food safety [3,4]. Numerous food safety-related incidents worldwide, such as the discovery of dioxin- and antibiotic-contaminated meats and pesticide residues in foods, have increased public distrust in food safety [3]. Food safety incidents undermine consumer trust in the food supply chain [5]. Consumers’ growing concerns about food safety have increased public demands for food safety accountability [6]. Food safety concerns motivate consumers to seek safer, more natural foodstuffs and avoid unhealthy, synthetic products. Consequently, consumers are increasingly requesting for more exhaustive details [7]—ranging from raw materials and ingredients to production methods, packaging and pesticide or hormone residue levels—in foods consumed daily [8]. Thus, the interest in improving food traceability systems for supply chains is increasing [7,9]. 

Food traceability is a systematic chain that can enhance food safety [10]. The European Union General Food Law Regulation (No. 178/2002) defines traceability as “the ability to trace and follow a food, feed, food-producing animal or substance intended to be, or expected to be incorporated into a food or feed, through all stages of production, processing and distribution” [11]. In academia, food traceability is defined as the essential information that can describe the processing history of natural food and any subsequent processes, including chemical components or ingredients added during the production processes [12]. Researchers have reported that the adoption of a food traceability system can reduce consumers’ perceived uncertainty and increase their purchase intention [4].

Food traceability and food traceability systems have been extensively studied. Kim and Woo [13] reported that the perceived ease of use and perceived usefulness of a quick response code for a food traceability system significantly influenced consumer intent to adopt the system. Shi et al. [14] proposed that consumers’ self-evaluation of health and awareness of food safety can influence whether they purchase certified traceable pork. Some studies have focused on the factors that affect consumers’ willingness to buy certified traceable food from the perspective of consumer characteristics such as education level and income [15] and level of food safety concern [16]. From another perspective, Chen et al. [17] investigated the role of product attributes (i.e., traceability information, price, appearance, and government subsidy) on consumers’ willingness to pay for certified traceable food.

Trust is a key factor that influences purchase behavior relating to foods [18,19], especially certified traceable foods [20]. Furthermore, institutional trust is the standard predictor of trust in major institutions in a ganizations [21], such as governments and legal organizations [22,23]. Institutional trust theory suggests that the people’s trust in an institution affects their perception of that institution [24]. Consumer studies have expanded institutional trust theory to explore the effects of institutional trust on perceived expertise [25], perceived benefit [26,27], perceived risk [27], product trust [25,28,29], interpersonal trust [28,30], willingness to participate in permission-based mobile marketing [22], loyalty in e-commerce [31], continuing intention to use e-government services, and online word of mouth [30].

Studies have assessed consumer trust in food systems and verified that it comprises trust in governments, producers, retailers [32,33,34], and certification organizations [33,35]. Chen [36] specified that industry-level trust comprises consumer trust in government and consumer associations, whereas firm-level trust comprises consumer trust in farmers, manufacturers, and retailers. Chen [36] also reported that trust in food manufacturers and retailers positively influences consumer perceptions of food safety but industry-level trust (i.e., trust in government and consumer associations) does not affect consumer perceptions of food safety.

Apart from food safety, food health benefit is also perceived as a key attribute that consumers relate to food traceability [37]. A food traceability system can increase consumers’ food purchase intention [4], and repeat customers are crucial to the success of a business [38]. However, few studies have explored the influence of institutional trust on health perceptions and repurchase intention pertaining to food, especially certified traceable fresh food. Thus, the following research questions are raised:

(1) Does institutional trust (i.e., trust in government, certification organizations, producers, and retailers) affect certified traceable fresh food consumers’ food safety perceptions, food health perceptions, and repurchase intention?
(2) Which types of institutional trust have a dominant effect on certified traceable fresh food consumers’ food safety and health perceptions and repurchase intention?

## 2. Literature Review and Research Hypotheses

### 2.1. Effect of Institutional Trust on Perception of Food Safety

Institutional trust refers to the fulfillment of consumers’ normative positive expectations by organizations [39,40]. Institutional trust is essential to relationships, certifications, and organizational assurances, and it can be promoted by increasing consumers’ confidence that normative and expected outcomes will be achieved [41]. Perceptions of food safety relate to a consumer’s concerns about whether a specific food can be consumed [42] without harmful effects [43]. For example, when selecting food, consumers may have specific concerns regarding safety, hygiene, cleanliness, and the presence or absence of chemical residues [44].

Consumers who purchase food usually interact directly with food retailers and indirectly with food regulators (who are responsible for managing food hazards) [36]; de Jonge et al. [32] suggested that consumers trust the organizations that form food supply chains (such as producers, manufacturers, and retailers of food products) and food regulatory institutions (such as governments, legislative institutions, and consumer associations). Previous studies have indicated that trust influences the perceived food safety of various food products [45]. Researchers have verified that trust in governmental associations influences consumers’ perception of food safety [46], and some have also reported that trust in food producers and retailers is positively correlated to consumers’ perceptions of food safety [36]. Thus, the present study proposed the following hypotheses:

**Hypothesis** **1a** **(H1a).** *Trust in government has a positive effect on consumers’ perceptions of food safety*.

**Hypothesis** **1b** **(H1b).** *Trust in certification organizations has a positive effect on consumers’ perceptions of food safety*.

**Hypothesis** **1c** **(H1c).** *Trust in food producers has a positive effect on consumers’ perceptions of food safety*.

**Hypothesis** **1d** **(H1d).** *Trust in food retailers has a positive effect on consumers’ perceptions of food safety*.

### 2.2. Effect of Institutional Trust on Perception of Health Benefits

Perceived health benefits refers to consumers’ interest in specific benefits that improve their physical health [47,48] and overall health [49,50] and reduce their risk of illness [50,51]. Studies have reported on the crucial relationship between trust and risk reduction [36,52]. Trust alleviates people’s concerns about risks [36]. Salazar et al. [53] suggested that increasing trust reduces perceived risk. Knight and Warland [54] also proposed that trust is a decisive factor in perceptions of food risk. Thus, the present study proposed the following hypotheses:

**Hypothesis** **2a** **(H2a).** *Trust in government has a positive effect on consumers’ perceptions of health benefits*.

**Hypothesis** **2b** **(H2b).** *Trust in certification organizations has a positive effect on consumers’ perceptions of health benefits*.

**Hypothesis** **2c** **(H2c).** *Trust in food producers has a positive effect on consumers’ perceptions of health benefits*.

**Hypothesis** **2d** **(H2d).** *Trust in food retailers has a positive effect on consumers’ perceptions of health benefits*.

### 2.3. Effect of Institutional Trust on Purchase Intention

Gilaninia et al. [55] defined purchase intention as the likelihood that consumers will purchase a specific product from within a product category in purchase situation. Diallo [56] described purchase intention as a consumer’s tendency to purchase specific products repeatedly and refrain from purchasing other products. Some studies have indicated that institutional trust has a significant and positive influence on behavioral intentions [22,30]. Therefore, the present study proposed the following hypotheses:

**Hypothesis** **3a** **(H3a).** *Trust in government has a positive effect on consumers’ purchase intention*.

**Hypothesis** **3b** **(H3b).** *Trust in certification organizations has a positive effect on consumers’ purchase intention*.

**Hypothesis** **3c** **(H3c).** *Trust in food producers has a positive effect on consumers’ purchase intention*.

**Hypothesis** **3d** **(H3d).** *Trust in food retailers has a positive effect on consumers’ purchase intention*.

## 3. Materials and Methods

### 3.1. Measurement

The measurements were conducted using a valid questionnaire employed in prior studies, with some of the descriptions from the original scale being slightly modified to be more suitable within the current context: The constructs of trust in government, trust in certification organizations, trust in producers, and trust in retailers were measured using four five-item scales developed by Chen [36]. Food safety perception was measured with a four-item scale from Seo and Yun [44], and food health perception was measured with a modification of Lee and Goudeau’s [57] four-item scale. Finally, repurchase intention was measured by employing a three-item scale adapted from Chih et al. [58]. The questionnaire’s measure items are listed in Table 1. The descriptions were measured by a 7-point Likert-type scale, ranging from 1 (strongly disagree) to 7 (strongly agree).

### 3.2. Data Collection and Sampling

Data were collected through paper-based surveys conducted at several traditional markets, hypermarkets, and supermarkets that sell fresh food with traceability labels. The participants were selected based on the proportion of the Taiwan population. The target participants had purchased certified traceable fresh food (e.g., fruits, vegetables, meat, and aquatic products) in the previous 3 months. A filter question was used to determine whether an individual corresponded to the profile of a target participant. The questionnaires were distributed in person to approximately 2000 target consumers, and 393 questionnaires were returned. Nearly 65% of the respondents were female; moreover, the largest age group was that of 21–30-year-old participants (30.5%), followed by 51–60-year-old participants (22.9%). Most (63.9%) of the participants had a college degree.

## 4. Data Analysis and Results

To calculate the constructs’ item reliability and validity, confirmatory factor analysis was used. As the factor loadings (λ) was required to be at least 0.5 [59], λ was 0.80–0.96 in this study (Table 1). Chau [60] suggested the standard of composite reliability (CR) should have a minimum of 0.8, and in the current study, CR was 0.89–0.90. Finally, Fornell and Larcker [61] suggested the average variance extracted (AVE) should be at least 0.5, and in the current study, AVE was 0.74–0.9, which indicated a strong convergent validity. As suggested by Fornell and Larcker [61], discriminant validity can be evaluated by comparing the correlation between two constructs with different square roots of AVEs. As presented in Table 2, each construct’s square root of AVE was higher than that of the respective correlation coefficients, indicating the magnitude of discriminant validity.

Multiple regression was performed to answer research question 1 and test the hypotheses. Hypotheses H1a–H1d predicted that institutional trust influences food safety perception. Our results, presented in Table 3, indicated that the four types of institutional trust, namely trust in government, certification organizations, food producers, and food retailers, were positively related to food safety perception; thus, H1a–H1d were supported. Hypotheses H2a–H2d predicted that institutional trust is positively related to food healthiness perception. Our results indicated that trust in certification organizations, food producers, and food retailers directly influenced food healthiness perception but trust in government was not a direct influence. Thus, H2b–H2d were supported but H2a was not supported. Finally, Hypotheses H3a–H3d predicted that institutional trust influences repurchase intention. Our results indicated that trust in certification organizations and food producers influenced repurchase intention but trust in government and food retailers did not significantly influence repurchase intention. Thus, H3b and H3c were supported but H3a and H3d were not supported. The explained variances of the institutional trust were 53%, 44%, and 30% for food safety perception, food health perception, and repurchase intention, respectively.

To answer research question 2, stepwise regression was performed to determine the type of institutional trust with the dominant effect on each of the outcome variables. As indicated in Table 4, the stepwise regression results suggested that four types of institutional trust significantly affected food safety perceptions (adjusted *R*^2^ = 0.53). In particular, trust in certification organizations enters the regression equation first in a stepwise regression and accounts for 43% of the explained variation in consumer food safety perceptions, followed in order of importance by trust in retailer, trust in producers, and trust in government. Taken together, these results indicated that the higher the institutional trust, the higher the food safety perceptions.

The results of the stepwise regression indicated that trust in certification organizations, trust in producers, and trust in retailers significantly affected food health perceptions, whereas trust in government did not have a significant influence on consumer food health perception. Trust in producers was entered first in the stepwise regression equation, and it alone accounted for 40% of the explained variance in consumer food health perception, followed in order of importance by trust in certification organizations and trust in retailer. Finally, trust in certification organizations and trust in producers significantly affected repurchase intention. Trust in producers was entered first in the stepwise regression equation, and it alone accounted for 28% of the explained variance in consumer repurchase intention, whereas trust in government and trust in retailers did not contribute significantly to the model and therefore were removed from the equation.

## 5. Discussion

With reference to institutional trust theory, the objective of the present study was to clarify the influence of consumer institutional trust (i.e., trust in government, certification organizations, food producers, and food retailers) on food safety and health perceptions and repurchase intention in the context of certified traceable fresh foods. The results indicated that trust in government, certification organizations, producers, and retailers significantly and positively influences food safety perceptions. Furthermore, trust in certification organizations, producers, and retailers is positively and significantly related to perceived health benefits of traceable fresh food, whereas trust in certification organizations and producers influences repurchase intention. In addition, whereas trust in certification organizations has a dominant effect on food safety perceptions, trust in producers is a dominant factor that influences consumers’ perception of the health benefits of certified traceable fresh food and their repurchase intention.

### 5.1. Theoretical Implications

Our results indicated that trust in government, certification organizations, producers, and retailers has significant positive effects on food safety perception. This is inconsistent with the findings of Chen [36], who reported that trust in food manufacturers and retailers has a positive influence on food safety perception, whereas trust in government and farmers is not associated with food safety perception. This inconsistency may be due to the differences in foods from various product categories (natural food vs. processed food). Specifically, the present study only examined certified traceable fresh foods, whereas Chen [36] investigated five processed food categories (i.e., edible oil, dairy products, bean products, cooked food, and infant food). In particular, no previous studies have examined the association of institutional trust with health perceptions and repurchase intention pertaining to food, especially traceable fresh food. The results of the present study indicated that for consumers of certified traceable fresh food, trust in certification organizations, producers, and retailers has a positive influence on their food health perception, whereas only trust in producers and certification organizations has a positive influence on repurchase intention. In summary, this research contributes to the literature by clarifying the relationships among institutional trust, food safety and health perceptions, and repurchase intention. 

### 5.2. Practical Implications

To conserve consumer food safety and health perceptions and repurchase intention, this study proposes the following recommendations to certified traceable fresh food marketers and related organizations. First, considering that the governments and certification organizations are the exclusively legislated forces that maintain order of the food traceability system, this study suggests that these organizations should formulate effective food tracing programs to build consumer trust and confidence. For instance, governments should strictly monitor the use of food traceability labels and enforce laws ensuring that producers and retailers comply with international and domestic food regulations. Second, certification organizations should ensure that all approved traceable fresh foods distributed in the food supply chains are safe to eat, meet hygiene standards (i.e., absence of infectious agents), and are supplied in sufficient quantities to meet nutritional demands. Third, food retailers should carefully screen traceable food providers. Fourth, consumer trust in producers is critical for their food safety and health perceptions and repurchase intention. Certified traceable fresh food producers should employ production processes that maximize food quality.

### 5.3. Limitations and Future Research Directions

This study has several limitations and suggestions for further research. First, in this study, convenience sampling was used, and sampling was restricted to Taiwanese cities. However, convenience sampling may lead to biased results that do not represent the entire population. In the future, study locations could be selected randomly. Second, the cross-sectional design of this study aided in collecting only the period-specific data. Additional longitudinal studies designed to detect causality are thus recommended. Third, the measurement of the food health perceptions was restricted to physical health and nutrition. Therefore, studies that include a more comprehensive set of food health perception measurements are warranted. Fourth, studies have highlighted multiple levels of institutional trust in the context of various countries and cultures [62,63]. To obtain clearer answers to our research questions, future studies should attempt to replicate our findings in other countries and social cultural settings. Fifth, the present study focused on certified traceable fresh food. Therefore, further research could include other certified food (e.g., health foods and organic foods) in cross-product comparisons to obtain valuable information that can serve as a reference for governments, certification organizations, and certified food marketers.

### 5.4. Conclusions

Due to several food safety incidents over the past 10 years, consumers have gradually lost institutional trust; this loss has led to an increase in consumer demand for certified food. To elucidate the role of institutional trust in traceable fresh food consumer behavior, the present study developed a survey that investigated the effect of institutional trust on food safety and health perceptions and repurchase intention. The results revealed that institutional trust has multiple effects on food safety and health perceptions and repurchase intention. With the increasingly essential role of traceable food in the food market, traceable food marketers must enhance their understanding of the behavior of traceable fresh food consumers.

## Figures and Tables

**Table 1:** Convergent validity tests results.

Variables	Observed Variables	λ	CR	AVE
Trust in government	The government has the competence to control the safety of traceable fresh food.	0.84	0.90	0.75
	Government agencies have implemented adequate rules and regulations to ensure traceable fresh food safety.	0.89		
	The government is concerned about the safety and health of traceable fresh food consumers.	0.88		
	If I were to encounter any problems with traceable fresh food quality or safety, government agencies would be available to provide assistance and support.	0.86		
	The government provides truthful information about traceable fresh food safety to consumers.	0.87		
Trust in certification organization	Certification organizations have the competence to guarantee the safety of traceable fresh food products.	0.87	0.89	0.76
	Certification organizations are concerned about the safety and health of traceable fresh food consumers.	0.92		
	Certification organizations pay special attention to the safety of traceable fresh food.	0.91		
	If I were to encounter any problems with traceable fresh food quality or safety, certification organizations would address these problems promptly and fairly.	0.83		
	Certification organizations provide truthful information about traceable fresh food safety to consumers.	0.82		
Trust in producer	Producers have sufficient knowledge and skills to guarantee the safety of traceable fresh food products.	0.84	0.89	0.75
	Producers always comply with regulations related to traceable fresh food safety.	0.89		
	Producers are concerned about the safety and health of traceable fresh food consumers.	0.91		
	When producers identify hidden safety problems in traceable fresh food production, they likely take the initiative to recall their products.	0.81		
	Producers are honest about the safety of traceable fresh food.	0.89		
Trust in retailer	Food retailers have sufficient knowledge and skills to guarantee the safety of traceable fresh food products.	0.84	0.90	0.76
	Food retailers always comply with regulations related to traceable fresh food safety.	0.91		
	Food retailers are concerned about the safety and health of traceable fresh food consumers.	0.92		
	If I were to encounter any problems with traceable fresh food quality or safety, food retailers would address the problems promptly and fairly.	0.80		
	Food retailers are honest about the safety of traceable fresh food.	0.90		
Food safety	Traceable fresh food is safe.	0.87	0.90	0.74
	Traceable fresh food is hygienic (e.g., free from pathogens or contaminants).	0.88		
	Traceable fresh food is clean (e.g., free from parasites or insects).	0.85		
	Traceable fresh food contains no chemical residues (e.g., free from pesticides or heavy metals).	0.84		
Health benefit	Traceable fresh food is nutritious.	0.88	0.90	0.81
	Traceable fresh food contains many nutrients (e.g., protein, vitamins, or minerals).	0.87		
	Traceable fresh food keeps me healthy.	0.93		
	Traceable fresh food is good for my body.	0.91		
Repurchase intention	I plan to continue purchasing traceable fresh food in the future.	0.95	0.90	0.91
	I intend to continue purchasing traceable fresh food in the future.	0.96		
	I expect to continue purchasing traceable fresh food in the future.	0.95		

Note: λ: factor loadings; CR: Composite Reliability; AVE: Average Variance Extracted.

**Table 2:** Correlations among latent variables.

Variables	Avg	S.D.	TIG	TIO	TIP	TIR	FS	HB	INT
Trust in government (TIG)	4.39	1.28	0.87						
Trust in certification organization (TIO)	4.96	1.18	0.75	0.87					
Trust in producer (TIP)	4.73	1.19	0.66	0.80	0.87				
Trust in retailer (TIR)	4.17	1.24	0.67	0.69	0.71	0.87			
Food safety (FS)	4.87	1.12	0.61	0.66	0.65	0.65	0.86		
Health benefit (HB)	4.86	1.18	0.48	0.62	0.63	0.54	0.66	0.90	
Repurchase intention (INT)	5.30	1.15	0.41	0.51	0.54	0.38	0.51	0.62	0.96

Note: Values highlighted in gray are the squared average variance extracted of each construct; the remaining values are the correlation coefficients between constructs, Avg: Average; S.D.: Standard Deviation.

**Table 3:** Results of multiple regression analysis.

Independent Variables	Dependent Variable		
	Food Safety	Health Benefit	Repurchase Intention
Trust in government	0.15 **	−0.6 ns	0.05 ns
Trust in certification organization	0.20 **	0.32 ***	0.21 **
Trust in producer	0.20 **	0.30 ***	0.38 ***
Trust in retailer	0.28 ***	0.14 *	−0.07 ns
Adj. *R*^2^	0.53	0.44	0.30

Note: ns, not significant; * *p* ≤ 0.05; ** *p* ≤ 0.01; *** *p* ≤ 0.001.

**Table 4:** Results of stepwise regression.

Model	Food Safety			Health Benefit			Repurchase Intention		
		Beta	Adj. *R*^2^		Beta	Adj. *R*^2^		Beta	Adj. *R*^2^
1	TIO	0.66	0.43	TIP	0.63	0.40	TIP	0.54	0.28
2	TIO	0.40	0.51	TIP	0.36	0.43	TIP	0.36	0.30
	TIR	0.38		TIO	0.34		TIO	0.22	
3	TIO	0.28	0.51	TIP	0.30	0.44			
	TIR	0.32		TIO	0.29				
	TIP	0.20		TIR	0.13				
4	TIO	0.20	0.53						
	TIR	0.28							
	TIP	0.20							
	TIG	0.15							

## Data Availability

The data in the presented study are available within the article and in the Supplementary Material.

## Supplementary Materials

The data are available at Figshare. (https://figshare.com/articles/dataset/Traceable_Fresh_Food/17061005, accessed on 17 November 2021).
